# Advancements in Assessments of Bio-Tissue Engineering and Viable Cell Delivery Matrices Using Bile Acid-Based Pharmacological Biotechnologies

**DOI:** 10.3390/nano11071861

**Published:** 2021-07-19

**Authors:** Armin Mooranian, Melissa Jones, Corina Mihaela Ionescu, Daniel Walker, Susbin Raj Wagle, Bozica Kovacevic, Jacqueline Chester, Thomas Foster, Edan Johnston, Momir Mikov, Hani Al-Salami

**Affiliations:** 1 The Biotechnology and Drug Development Research Laboratory, Curtin Medical School, Curtin Health Innovation Research Institute, Curtin University, Bentley, Perth, WA 6102, Australia; a.mooranian@curtin.edu.au (A.M.); melissa.a.jones@student.curtin.edu.au (M.J.); c.ionescu@postgrad.curtin.edu.au (C.M.I.); danieljcswalker@gmail.com (D.W.); susbinraj.wagle@postgrad.curtin.edu.au (S.R.W.); bozica.kovacevic@postgrad.curtin.edu.au (B.K.); j.chester@graduate.curtin.edu.au (J.C.); thomas.p.foster@student.curtin.edu.au (T.F.); edan.johnston@student.curtin.edu.au (E.J.); 2 Hearing Therapeutics, Ear Science Institute Australia, Queen Elizabeth II Medical Centre, Nedlands, Perth, WA 6009, Australia; 3 Department of Pharmacology, Toxicology and Clinical Pharmacology, Faculty of Medicine, University of Novi Sad, Hajduk Veljkova 3, 21101 Novi Sad, Serbia; momir.mikov@mf.uns.ac.rs

**Keywords:** bioartificial organ, cell microencapsulation, immunogenic considerations, microcapsule analysis, in vitro testing

## Abstract

The utilisation of bioartificial organs is of significant interest to many due to their versatility in treating a wide range of disorders. Microencapsulation has a potentially significant role in such organs. In order to utilise microcapsules, accurate characterisation and analysis is required to assess their properties and suitability. Bioartificial organs or transplantable microdevices must also account for immunogenic considerations, which will be discussed in detail. One of the most characterized cases is the investigation into a bioartificial pancreas, including using microencapsulation of islets or other cells, and will be the focus subject of this review. Overall, this review will discuss the traditional and modern technologies which are necessary for the characterisation of properties for transplantable microdevices or organs, summarizing analysis of the microcapsule itself, cells and finally a working organ. Furthermore, immunogenic considerations of such organs are another important aspect which is addressed within this review. The various techniques, methodologies, advantages, and disadvantages will all be discussed. Hence, the purpose of this review is providing an updated examination of all processes for the analysis of a working, biocompatible artificial organ.

## 1. Introduction

Microencapsulation presents a possible method that may be useful in the production of bioartificial organs. Encapsulation provides a selectively permeable barrier whilst protecting the encapsulated contents from the host’s immune system. This membrane allows nutrient and waste exchange [1]. This is why there is considerable interest in microencapsulation in relation to the implantation of bioartificial organs or microdevices.

Working, safe bioartificial organs provide the potential for treatments for a variety of disorders. Consequentially, they have been investigated for the creation of a wide range of transplantable organs including, but not limited to the kidney, heart and liver [2]. Microencapsulation of cell lines and islets also offer the potential for use in a bioartificial pancreas, something which has garnered significant interest [1]. Therefore, islet and cell line microencapsulation will form the primary example of this technology’s potential applications in this review.

A multitude of methodologies will be discussed and outlined within this paper in order to provide an updated review of such, including outlining the purpose, benefits, techniques and any disadvantages or limitations of each. Stepwise, the basics must be assessed and evaluated first, hence why analysis of a microcapsule is imperative for such development of an organ. The microcapsule’s physical and chemical properties must be evaluated, with techniques such as microscopic analysis, and spectroscopies discussed in detail [3]. Furthermore, the analysis of a microcapsule’s composition and mechanical strength are important properties which must be assessed [4]. Methods to do so are included.

Following the assessment of the microcapsule properties, the encapsulated cells must be analysed. These islet and alternative cell lines must be cultured, various techniques to do so will be discussed. Following encapsulation, these cells should be assessed, with techniques to examine their activity and survival imperative [5].

In addition to capsule and cell methodologies, immunological considerations must be accounted for if the organ is to exist within a living system. The combination of these tests should indicate the compatibility and survivability of such an organ post-transplantation, and must be accounted for in studies of the development of bioartificial organs [6]. Therefore, such considerations and analysis of such will be included in this review.

Overall, a successfully implanted bioartificial organ must have idealistic physical and chemical properties as well as biocompatibility [7]. Hence, a wide range of technologies and assays must be utilised to characterise and evaluate the artificial tissue, and as such and will be explored throughout this review, including immunogenic considerations in such a context.

## 2. Microcapsule Analysis

### 2.1. Microscopic Analysis

**Figure 1:**
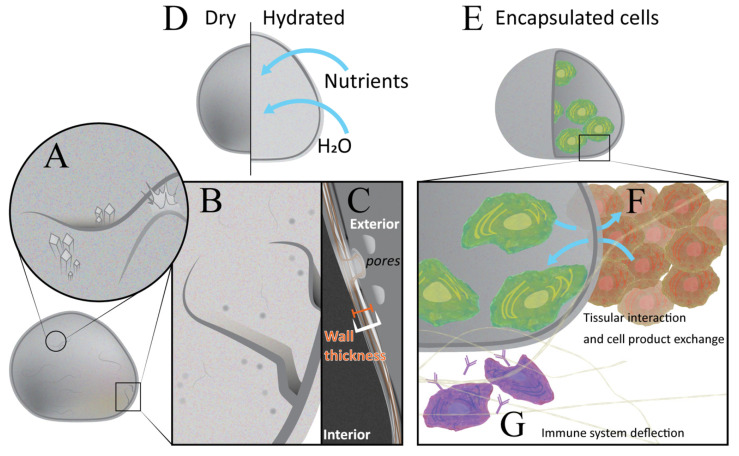
Example of a microcapsule and properties for analysis. (**A**) Microscopic view of the surface of a microcapsule. (**B**) Microcapsule pores and cracks which may be viewed via analysis described in the review. (**C**) Further zoomed view of the microcapsule wall and pores. (**D**) Comparison of a dry and hydrated microcapsule following nutrient and water addition. (**E**) Internal view of a microcapsule showing the encapsulated cells within. (**F**) Magnification of the encapsulated cells showing their interaction and cell product exchange, which may be assessed via methodology outlined within this review. (**G**) Schematic of immune system deflection which is imperative to a successful bioartificial organ and for microcapsules containing cells. Such immunogenic considerations are highlighted in this review. All elements indicated in this schematic can be examined preliminarily via methodology outlined within this review prior to in vivo studies.

In order to analyse the appearance of the inner contents of the microcapsule, including cells, optical microscopy is commonly used [3], see Table 1 for a comparison of each technique discussed. An example of how microscopy can be used in the context of microcapsule visualisation can be seen in Figure 1, panels A,B,C,F. The microcapsule’s diameter and wall thickness can be determined and measured under light microscopy, with this technique also offering the ability to analyse the overall topography and uniformity of microcapsules, as seen in Figure 1**,** panel C [8,9]. Capsular geometry is important for the prediction of biocompatibility and in vivo behaviour, as sphericity is a paramount requirement of capsule stability and uniformity; hence why techniques such as microscopy are greatly advantageous in the early stages of testing [10]. Furthermore, the screening of bacterial and/or fungal overgrowth in the culture media used to grow the cells can be conducted routinely or on a daily basis with the aid of light microscopy [8].

Confocal laser scanning microscopy (CLSM) is a useful technique commonly employed to analyse the distribution of polymers and cross-linking ions inside microcapsules [11]. This method of analysis is used in cell encapsulation research as it can illustrate the homogenous or inhomogeneous nature of polymer and/or co-polymer distribution within the microcapsules. In addition, such a technique can provide an indication of any polymer-excipient interactions, as well as potential permeability of the surrounding membrane [12,13]. In terms of traditional CLSM on live cells, the technique does have a significant disadvantage, it causes phototoxicity. Furthermore, in comparison to other, newer models, the speed and sensitivity ratio for imaging is poor [14].

The distribution, density and growth patterns of encapsulated pancreatic islet cells can also be determined using CLSM via staining of the cells with dyes such as fluorescein diacetate which stains live cells green, and propidium iodide which stains dead cells red. CLSM may also be combined with flow cytometry to determine the extent of cellular proliferation within the microcapsules before and after transplantation using viability assessment kits containing carboxyfluorescein succinimidyl ester (CFSE) [15,16,17,18]. Mooranian et al. conjugated the bile acid ursodeoxycholic acid (UDCA) with the fluorescent dye tetramethylrhodamine (TRITC) to form TRITC-UDCA conjugates and applied CLSM technology to study the partitioning of the bile acid UDCA within their microcapsules [5]. UDCA is a dihydroxy secondary bile acid which is a highly hydrophilic bile acid which has been explored in drug delivery. UDCA is produced via the 7β epimerization of primary bile acid chenodeoxycholic acid, with the chemical structure 3α,7β-dihydroxy-5β-cholan-24-oic acid [19,20]. The authors were able to show that the bile acid homogeneously disperses within all the layers of the microcapsules and is in direct contact with the encapsulated β-cells, influencing their viability and functionality, as demonstrated via MTT assays and bioenergetic/metabolic profiling, two techniques that will be discussed further in this review. Information such as this is invaluable to researchers for future studies, and was achieved via utilisation of a common technique [5], see Table 2.

Scanning electron microscopy (SEM) is also often used to accurately visualize and characterize the surface structure, overall geometry and surface composition of the microcapsules, as applied to a microcapsule as visualized in Figure 1 [21]. Due to the high levels of image quality now available with SEM, it can give an insight into the porosity, permeability and rigidity of the surface of any test subject microcapsules, and can also provide detailed 3D images of the capsules [22]. Therefore, SEM has the ability to provide important information regarding the topography and morphology of the microcapsules. This is particularly significant as surface structure and composition is among the critical requirements for a successful microencapsulation system to be developed [23,24]. It should be noted that the harsh conditions deployed using standard SEM machinery to analyse hydrogel-based microcapsules have the potential to result in dehydration and deformity, giving rise to artefacts [25]. In an attempt to reduce these impacts, environmental SEM may be utilised [26].

Electron dispersive X-ray spectroscopy (EDXR) is often used in tandem with SEM, as most modern instruments can generate both sets of data within the one piece of equipment. The use of EDXR is vital for the analysis and subsequent surface characterization of the examined microcapsules. This is beneficial because it allows the elemental characterization of the surface to be reported. Furthermore, quantitative information regarding the elemental composition of the surface can be achieved as the number and kinetic energy of reflected electrons is in direct proportion to the number of atoms from the element they originate from. Built-in software can provide information on both atomic percentages and weight percentages [26]. Negrulj et al. applied EDXR surface analysis to hydrogel-polyelectrolyte microcapsules in order to study the influence of UDCA on both the surface morphology and atomic makeup of the microcapsule surfaces [27]. In alternatives studies, researchers were able to complement their findings with Fourier transform infrared spectroscopy (FTIR) studies, which will be discussed further, to show UDCA’s chemical compatibility with the other excipients forming the microcapsule surface [5].

EDXR does have limitations, including that it only indicates surface elemental composition; whereas it is often the case that the chemical groups below the surface of the microcapsules are also vital to assess. These deeper chemical structures are important as they reveal more information about chemical bonds that are forming within the capsule [28,29,30]. Therefore, other techniques such as FTIR have been deployed to examine these interactions.

### 2.2. Physical and Chemical Analysis

FTIR is an analysis which looks at the surface of microcapsules on a micrometre scale, rather than simply providing atomic composition and quantification; it provides an absorption spectrum of the sample, offering data on the nature of chemical groups present on the surface [31,32,33]. As an example, FTIR has been applied to show that interactions between polyelectrolytes, in this case poly-l-lysine (PLL), and alginate are based on hydrogen bonding. This was demonstrated as FTIR surface data, when referenced against calcium-alginate microcapsules, allowed researchers to conclude that PLL displaced Ca^2+^ upon binding to the alginate surface via hydrogen covalent bonding [34].

FTIR has also been utilised in studies of microencapsulated islets to demonstrate chemical composition, including of the microcapsules without the cells [35,36]. Most often, in the field of islet encapsulation, attenuated total reflectance (ATR), forming ATR-FTIR is deployed as it provides an analytical penetration depth of 0.2–2 µm allowing for multi-compartmental analysis within the microcapsule structure to take place [37,38,39]. FTIR does also have its disadvantages, including that only thin samples, often only up to 20 μm, can be analysed using this technique. This indicates that samples, including microcapsules, must be crushed for analysis and therefore, destroyed, hence preventing those analysed being used in future studies [31]. It is important to note that accurate surface composition acquisition is not merely limited to one instrument or technique, as the data obtained from EDXR and FTIR are not substitutive but complementary in nature, providing a more complete data sequence [40].

Differential scanning calorimetry (DSC) is an important technique utilised for the thermal analysis of chemical compounds as well as intact microcapsules. This category of thermal analysis is useful in the field of Artificial Cell Microencapsulation (ACM) as it allows the determination of how the physical characteristics of a substance change along with temperature over a certain period of time [41]. It allows the user to determine the thermodynamic state of the material being examined, and whether the excipients exist in amorphous, crystalline or unstable polymorphic form in the microcapsule system [42]. Thus, it permits the investigator to determine the thermal stability of the microencapsulation system being applied and the nature of any interaction amongst excipients, as well as analysing whether polymorphism exists in the system [43]. This has important ramifications as excipient incompatibility or structural lattice changes that take place from the pharmaceutical dispersion system to the microcapsule formation in situ could give rise to instability over the duration of an implanted microcapsule system [41]. However, DSC does have limitations, including the need to avoid any bubbles which would invalidate results. The capillary size indicates the system is prone to such bubble formation. When used for water and hydrate analysis, overlapping hydration peaks limit DSC functionality [44].

Another area that should be tested is the porosity and swelling characteristics of the microcapsules. This can be assessed via the placement of a predetermined number of microcapsules in an aqueous buffered solution. This method is limited as it relies upon weight changes to indicate a swelling index. Swelling studies of microcapsules is an important assessment as mechanical disintegration of the hydrogel network by water molecules leads to increased permeability, which can result in exposure to labile cells within the microcapsules and, therefore, trigger the immune system [45,46]. This is conducted at temperatures and conditions mimicking the expected physiological parameters at the site of implantation or delivery and potential route to arrive at such location [27,47,48]. Similarly, swelling of alginate-containing microcapsules may lead to the release of cellular components which can also trigger an immune response. Additionally, Ca^2+^ leakage from Ca^2+^-alginate microcapsules following degradation of the bead membrane integrity has been shown to enhance dendritic cell activity, giving rise to detrimental graft failure and rejection [49,50].

Mechanical strength of microcapsules under pH or osmotic stresses can be measured by agitating vials of microcapsules in sodium chloride or phosphate buffer solutions. Placement of the vial in a shaker and vibrating at a specific frequency at various time intervals, allows the number of fractured capsules to then be counted or weighed and compared to the starting number or weight to determine an overall spectrum of mechanical strength and swelling amongst tested subjects. To further quantify this, the microcapsules may be placed in phosphate buffers of various pH values and at various temperatures to stimulate transplantation. However, this technique does have disadvantages, such as relying upon visual inspection and microscopy to compare the broken or damaged capsules to their original condition and differentiate their properties. In addition, size changes and buoyancy can also be assessed using this methodology [51,52].

Mathavan et al. conducted mechanical strength studies via placement of microcapsules on a multishaker and recording microcapsule damage data over time intervals. Such studies allowed the authours to assess the microcapsule’s properties and impacts from pressure and stress conditions. Within the investigations, the authours determined that the addition of bile acid taurocholic acid to gliclazide-sodium alginate microcapsules improved the mechanical strength, with microcapsules maintaining shape and drug contents to a statistically significant value from their control subject [52].

A homogeneous particle size distribution is critical for ensuring consistency in the microcapsules produced and ensures uniformity in their encapsulated contents. Figure 1 a microcapsule which can be assessed for uniformity and size. Some methods used to determine the average particle size for a set of microcapsules typically include microscopic analysis and the use of particle size analysis instruments [53,54].

To further analyse the surface property of the microcapsules and investigate their stability for in vivo applications, it is necessary to determine the surface charge of the capsules. This provides an indication of the electrokinetic potential of a particle. This measurement can be used as an indication for the interaction between the microcapsule and surrounding tissues [55,56]. It can be measured in the same way as zeta potential using a zeta sizer. Ideally, the surface charge of microcapsules containing enclosed islets should be negative and on par with the cell membrane potentials of adjacent cells. This is because a positive surface charge promotes protein adsorption and subsequent activation of the immune system, something to be avoided. The surface charge of a microcapsule is usually measured via the electrophoretic mobility of an electrolyte solution containing the microcapsules which is pushed through a measuring cell, with this resulting in a pressure drop proportional to the flow resistance across the cell [25]. The movement of circulating electrolytes through the measuring cell generates the flow of ions, and the subsequent potential difference is detected. This detected voltage corresponds to the actual charge on the surface of the microcapsules [55].

Studies by de Vos et al. conducted studies of zeta potential on alginate-PLL capsules, finding results which were comparable with biological assays conducted post-implantation of the capsules within a rat. Such results indicate the importance of pathophysiological assessments such as these zeta potentials in providing indications of how capsules will perform in vivo [55].

Direct measurement of the surface charge is impossible given the size of the microcapsules produced, thus the aforementioned zeta potential method is used [25]. It is also worth noting that the surface charge on the microcapsules is dependent upon the characteristics of the microenvironment fluid system in which they are placed. This is because the pH and ionic strength of the surrounding fluid impact the surface charge of the microcapsules. For instance, the zeta potential of islet containing alginate microcapsules would differ depending upon whether they are implanted intraperitoneally, under the kidney capsule or adjacent to the liver. Therefore, zeta potential measurements should be measured under conditions analogous to the physiological state, otherwise incorrect assumptions would arise surrounding stability based on surface charge characteristics [55,57].

Given the size of islet containing microcapsules (typically within a range between 300–800 µm) analysis of their surface charge using standard analytical equipment can be problematic [25,55]. Measuring the electrokinetic potential of the pharmaceutical dispersion system pre-encapsulation would give an indication of the formulation stability. This is particularly useful if detecting alterations after incorporation of new excipients to the existing system or analysing changes to pre-existing ingredient concentrations. However, it is important to note that this does not directly correlate to the actual surface charge of the final, formed microcapsules at the transplantation site. The results obtained are only valid for pharmaceutical formulation stability testing pre-encapsulation and for future manufacturing considerations. This is due to the fact that the greater the magnitude the zeta potential of the formulation liquid dispersion system is, the more stable the system and subsequently, there is a reduced risk of particle flocculation/aggregation and sedimentation over time [45,48,55,58,59].

A feasible viable cell delivery system must first meet several essential requirements; particularly, that it must be a stable and reproducible system with optimal characteristics for in vivo applications and potential large scale pharmaceutical manufacturing [60,61,62,63].

Stability can be defined in several ways and is often categorized as either biological or physical/chemical. Biological stability is centred on the performance of the microcapsules in vivo and could be tailored around immunogenicity, which encompasses both biocompatibility and bio-tolerability and is discussed further below; or activity around the implantation site [25]. Specifically, it has been found that alginate-based microcapsules have profoundly different stability profiles depending upon where they are transplanted. Using FTIR, surface analysis was carried out to determine the relative proportion of outer layer alginate to middle layer polyelectrolyte (in this case poly-ornithine) at various surgical transplantation sites including the brain, subcutaneous skin and peritoneum. The authors were able to conclude that the microcapsules were stable for at least 6 months when injected into subcutaneous tissue or into the brain. There was however a rapid degradation of the microcapsule’s membrane at the peritoneum, suggesting that modifications to the original formulation would be required if intending to target that site [64].

On the other hand, physical/chemical stability is concerned with the mechanical strength of the microencapsulation system and is determined via a series of experiments which include the previously described swelling and porosity, surface charge potentials, mechanical resistance (via a shaker or vortex), as well as the effects of temperature and pH on the integrity and performance of the microcapsules [58,65].

Two of the most important factors influencing the transplantation site for microencapsulated pancreatic islets and β-cells are the mechanical strength of the microcapsules and the biocompatibility at the transplantation site [60,66]. As seen in Figure 1, the microcapsule must be able to deter immune cells activation whilst maintaining adequate nutrient and waste exchange. Target sites include the intraperitoneal cavity, bone marrow, subcutaneous tissue, omentum pouch and under the kidney capsule [67,68,69,70]. The intraperitoneal cavity site results in the poorest encapsulated cell functionality because of profound inflammation, relatively poor vasculature and low implantation volume. For transplantation studies involving rodents, implantation subcutaneously or under the kidney capsule have been proven to be the most optimal due to a decreased immune response in comparison to intraperitoneal transplantation [71]. In the case of clinical human islet transplantation, intraportal hepatic infusion is most preferred due to high access to systemic circulation. This transplantation site has been shown in clinical trials to result in very high success rates, as measured by recipient insulin independency, without the need to use immunosuppressive drugs. However, a major drawback with intraportal hepatic infusions in human subjects is the risk of thrombogenesis stemming from immune system activation from the microcapsules. This may result in the los off 50% of viable transplanted cells. Therefore, there has been focus on finding alternative transplantation sites for human clinical trials [72,73].

## 3. Examination of Microencapsulated Islets and Cell Lines

### 3.1. Islet and Cell Line Culture

Microencapsulated pancreatic islets and insulinoma cell lines can be cultured using traditional media such as Roswell Park Memorial Institute (RMPI) 1640, Connaught Medical Research Laboratories (CMRL) 1066 and Dulbecco’s Modified Eagle’s Medium (DMEM), making them more versatile [5,63,74,75]. From these aforementioned media, CMRL has been found to be effective for culturing islets in vitro, although developments continue to be made to find more optimal media combinations [76,77,78]. The CMRL media is supplemented with fetal bovine serum (heat inactivated at 10% *v*/*v* concentration), glucose (5.6 mM) and antibiotics such as penicillin or streptomycin for optimal viability and proliferation [76]. If necessary, 10 mmol/L HEPES buffer can be added to stabilize the media against pH changes [3,79]. Often, it is required to also supplement the alginate-polyelectrolyte solutions, prior to microencapsulation and cell incorporation, with glucose, amino acids, insulin transferrin or other nutrients. This supplementation is important, as this has been found to enhance cell viability and functionality post-microencapsulation. In addition, it has been demonstrated that enhanced islet and β-cell performance may be attained via the incorporation of epithelial growth factor at 100 ng/mL to the culture media. Other media supplements investigated include nicotinamide (2–5 mM, prevents islet necrosis), antioxidants (ascorbic acid), selenium (6.25 ng/mL, involved in antioxidant enzyme activity), magnesium (5.3 mM, involved in insulin biosynthesis) and cysteine (1 mM, prevents oxidative damage) [76].

Post-microencapsulation and transplantation, it is common practice to remove the microcapsules in order to study their physicochemical characteristics, morphology as well as the viability of the islets and cells within them [74,75]. Such cells within a microcapsule may be seen in Figure 1. Most of these assays and examinations are straightforward, as they involve examining and analysing intact microcapsules. However, to definitively confirm pancreatic β-cell viability and activity prior to, during and post-encapsulation, it is necessary to evaluate “free” cells [74]. This often means the microcapsules need to be ruptured or “decapsulated” to release the islets and cells so they may be re-cultured. After re-culturing further investigation can be carried out. Cells can be assayed for viability, degree of potential proliferation and growth as well as biological activity [74,75]. Should the hyperglycemic state return in the transplanted recipients post-graft removal, this then adds further evidence to the clinical performance of the microencapsulated pancreatic cells [80,81].

A variety of chemical reagents can be used to culture islet/cell containing microcapsules for short periods of time in order to gently rupture the islets/cells [82,83,84]. The common method is to place a set number of microcapsules (routinely around 50–100) into a sterile container containing 1 mL PBS or culture media, be it RPMI, CMRL or DMEM, supplemented with 0.25% trypsin and 0.5 mM EDTA and incubating at 37 °C CO_2_ (95% humidified air and 5% CO_2_) with gentle shaking periodically [74]. After rupture, islets/cells are washed with fresh media and cultured as monolayers in tissue/cell culture flasks where assays for viability, such as MTT or via viability staining using dyes such as 6-carboxyfluorescien diacetate, (FDA); can be conducted. Alongside these assays, biological function assessments such as secreted insulin content and metabolism/bioenergetics of the islets and cells may also be carried out [74,75]. These techniques are explained in greater detail below.

### 3.2. Islet and Cell Line Examination

The analysis of microcapsules containing pancreatic islets is often characterized by two main techniques, either physicochemical or in vitro (biological). Within each of these two main groups are a series of analytical experiments which aid in the overall characterization of the microcapsules and each of these techniques is discussed in detail below. An overall summary of each technique can be found in Table 2. 

In terms of microencapsulated cells, various assays may be conducted to measure and quantify the cell viability and the metabolic activity. One calorimetric method for determining cell viability is the MTT assay, which, without destroying the microcapsules, can measure cell viability. In summary, this method utilises the MTT reagent, 3-(4, 5-dimethylthiazol-2-yl)-2, 5-diphenyltetrazolium bromide (MTT), which, when undergoing a reaction with viable cells, forms purple formazide. This is subsequently dissolved with solubilizing agents, including dimethylformamide and DMSO. In the event of cell death, cells cannot convert the MTT to the purple formazide. The change in colour (if present) can be measured photometrically [85,86,87]. Despite its ability to determine viability, the MTT assay does have drawbacks, including that microcapsules put through the assay are no longer viable, and the that increased incubation with MTT reagents causes improved sensitivity in results, but is more cytotoxic [84]. Despite known limitations, the MTT assay is still commonly used in the field of islet cell microencapsulation [5].

To improve the MTT assay, other tetrazolium reagents that reduce to formazan products have been investigated to assess cell viability. These reagents include 2,3-Bis-(2-Methoxy-4-Nitro-5-Sulfophenyl)-2*H*-Tetrazolium-5-Carboxanilide (XTT) and 3-(4,5-dimethylthiazol-2-yl)-5-(3-carboxymethoxyphenyl)-2-(4-sulfophenyl)-2H-tetrazolium (MTS). One advantage of these assays is that no second reagent is required for solubilization of the formazide, allowing readings to be taken periodically [87,88]. Similarly, negatively charged 2-(4-iodophenyl)-3-(4-nitrophenyl)-5-(2,4-disulfophenyl)-2H-tetrazolium) (WST-1) is reduced to another variation of water-soluble formazide. However, WST-1 is unable to penetrate the cell, so the reduction reaction occurs outside, making it unsuitable for widespread use in microencapsulated cells [88,89].

Resazurin, or Alamar Blue, is a water-soluble indicator which can be utilised to assess cell viability. Studies have indicated it is more sensitive than the tetrazolium-based assays, as well as better at being able to be used homogenously with cells. In a study of microencapsulated cells, Xiao et al. found that resazurin was comparable to the traditional MTT assay in terms of cell viability measurement as well as being a non-invasive process and not requiring the rupture of microcapsules [90,91,92]. Despite their promise, the cytotoxic nature of WST-1 and resazurin still makes them unviable for monitoring cell viability [89].

Additional methods are required to quantify the cells and assess cell proliferation as the MTT, and similar assays do not provide this information. These methods include dye exclusion tests, whereby the membranes of living cells are intact, which prevents them from taking up dyes, whereas broken membrane of dead cells will take up these dyes. Such a membrane can be visualised in Figure 1. Dyes that may be utilised in such assessments include eosin, trypan blue and propidium [93,94].

The most used method is the trypan blue assay; cells in PBS suspension have trypan blue dye added. The principle is that cells that take up the dye and possess a blue cytoplasm are nonviable, whereas the viable cells exclude the dye, and, therefore, have a clear cytoplasm. The cells are then assessed visually, typically with the aid of microscopy. This method has its pitfalls, including the subjective nature of such a test, whereby minimal dye uptake may not be accounted for, something which is of importance when exact numbers are required. Furthermore, viable cells are determined as those which do not take up the dye, however, exclusion of dye only indicates an intact membrane, not the function or growth ability of the cells. One proposed technique to overcome the subjective nature of this assay is to utilise flow cytometry to obtain more precise measurements [93,94]. One key limitation is that, in terms of utilisation of this method in microencapsulated cells, rupture and dissolution of the microcapsules is required in order to quantify viability [91,92].

Viability of encapsulated cells may be determined using fluorescence stains such as carboxyfluorescein succinimidyl ester (CFSE). Viable cells’ nuclei will be stained with this technique, which can then be visualised utilising fluorescence microscopy [95,96]. CFSE stained cells also have the potential to be quantified using flow cytometry or with a fluorescence-activated cell sorter. One advantage of this stain is that in appropriate concentrations, it possesses no adverse impacts on the cell [97]. One key disadvantage of CFSE staining is that in high concentrations, the dye is toxic to the cells it stains, with some cell types such as lymphocytes being very sensitive to such toxicity [98].

In terms of in vivo assessment of cell viability and quantification, one study by Chan et al. suggested a pH-nanosensor-based magnetic resonance imaging (MRI) technique. This study used encapsulated human hepatocyte cells with low-viscosity alginate, with several formulations, including alginate-PLL examined. This assessment technique may characterize cell death in a non-invasive manner, assessing the pH changes which are clinically indicative of cell death. The authors indicate that their study may be replicated for use in all instances with hydrogels due to the clinical grade of instrumentation and technique [99].

Another important method to determine cell viability and functionality is encapsulated cellular proliferation, with this analysis playing a crucial role in the success of a biologically functioning artificial organ [100]. To determine proliferation rates, 5-bromo-2-deoxyuridine (BrdU), a thymidine analogue, can be incorporated into replicating DNA. This can be conducted and quantified using flow cytometry, or ultrasensitive enzyme linked immunosorbent assay (ELISA) techniques in order to determine the cell cycle status of cells being analysed [101]. BrdU is a mitotic marker that is taken up by dividing cells in the S phase of mitosis, which is essential for DNA replication [102]. In comparison to other mitotic markers such as Ki-67 or proliferating cell nuclear antigen, BrdU is retained in cells independent of the cell cycle phase, resulting in detection in post-mitotic cells. BrdU staining is often carried out in order to determine the extent of cellular proliferation and differentiation of encapsulated foetal islet and stem cells [100].

BrdU staining does have limitations, including that the assay requires strong denaturing conditions, which both degrades the specimen and indicates that staining intensity is wholly dependent upon these denaturing conditions, and hence the subsequent results [103]. Kedziorek et al. used BrdU staining to demonstrate that microencapsulated mesenchymal stem cells maintained capacity to undergo replication in vitro [104].

As the aim of encapsulated pancreatic islet cells is to create a bioartificial pancreas when transplanted in vivo, it is critical to test for insulin content under static glucose stimulation challenge tests to illicit the biological activity of the encapsulated islets. This is typically done by exposing the microcapsules to various glucose concentrations, to mimic the basal or stimulated insulin release in response to glucose, for a set period of time. In order to achieve this, it is conducted under experimental parameters resembling the expected physiological conditions. The secreted insulin is then measured by radioimmunoassay or ELISA [105,106].

## 4. Immunogenic Considerations

### 4.1. Role of Immune Cells

The potential immunogenic properties of islet-containing microcapsules must be taken into consideration and assessed in order to proceed with transplantation [6]. Methods have subsequently been developed to characterise, in vitro, immunogenic properties. These assays have the potential to be tailored to analyse the immunogenicity of microcapsules. One of these methods utilises human whole blood models to assess blood-mediated inflammatory responses. This is done to allow for the analysis of interactions with complex cells, biomarkers and cascade systems present in blood, which are capable of coagulation and complement activation from the introduction of a foreign body, such as transplanted microcapsules. The instant blood-mediated response results in both thrombotic and inflammatory action, potentially resulting in the loss of transplanted material; hence the use of such models to exploit a predictive method for biocompatibility issues should be a requirement prior to assessment in animal models [107,108,109].

Another method to assess immunogenicity and cytotoxicity is in vitro assessment in appropriate cell lines [110,111]. This technique involves culturing cells alongside produced microcapsules, to elicit inflammatory responses due to the microcapsule constituents which are in contact with the cells. The cytokines produced the incubation can then analysed via assays such as cytometric bead arrays (CBA) [89,112,113].

Finally, some authors favour co-culturing of cells as the best method to detect and quantify any immune response from microcapsules, as the immune system is a complex inter-connected network of cells and mediators working in conjunction to produce an inflammatory response; it is not merely a single cell-type reaction. As a result, co-culturing macrophages with lymphocytes is becoming increasingly popular, and it has been found to be a good predictor of immunogenicity and inflammatory reactions closely mimicking in vivo responses [112,114,115].

### 4.2. Islet Derived Cytokines

Immune system activation and cytokine production by dendritic cells and macrophages upon contact with alginate-based microcapsules is well documented. However, more recently it has emerged that islets themselves secrete pro-inflammatory low molecular weight biomarkers. These biomarkers have been found to be capable of penetrating through the microcapsule membrane and triggering inflammation by recruiting and activating circulatory immune cells [113,116,117,118]. In particular, isolated pancreatic islets have been shown to secrete TNF-α, IL-6, IL-1β, IL-8, MCP-1 and MIP-1α, as well as nitric oxide (NO) both in vitro and in vivo; all of which trigger inflammatory reactions that may lead to transplantation failure [117]. Interestingly, islets have been shown to secrete more pro-inflammatory cytokines under stresses such as those arising from isolating and preparing them pre-transplant, and the conditions of the surrounding environment such as inflammation at the site of transplantation [117,119]. In addition, islet-derived cytokine levels increase with increasing glucose levels during in vitro culturing, as well as in vivo hyperglycemic states, and this suggest islets themselves play a key role in glucose-induced inflammation associated with diabetes mellitus development and propagation [120].

Reducing levels of pro-inflammatory cytokines derived from encapsulated islets is, therefore, just as important as efforts implemented in order to create biocompatible, non-immunogenic hydrogel-polyelectrolyte microcapsule membrane systems [113,116,117,121]. Bile acids have been found effective in reducing inflammation. For example, UDCA has been shown to be effective in reducing inflammation at the site of surgery [59,113,116,117,121,122]. Promising results have also been demonstrated to be attained via pre- and post-transplant administration of anti-inflammatory compounds, such as aspirin, in animal studies; as well as immunomodulatory agents, such as cyclosporine, for short durations. However, these medications have potentially serious side effects. They possess the potential to exhibit an array of drug interactions in a patients, so therefore, risks and benefits must be carefully considered [117]. The problems surrounding immunosuppressive drugs could be overcome with the use of anti-inflammatory peptide conjugated alginates which may be able to play a role in down regulating immune reactions towards hydrogels used for islet encapsulation [123]. Research in other areas has found conjugated alginates are able to assist in the suppression of immune reactions; techniques which may be useful in islet encapsulation [124,125].

Other techniques have been assessed to enhance islet transplant survival via the reduction of pericapsular fibrotic overgrowth, in which fibrotic tissue surrounds implanted microcapsules, resulting in limited graft survival. These include co-encapsulation in which companion cells possessing immunomodulatory properties are included within the produced microcapsule. One example is Sertoli cells, which may improve overall graft survival due to their ability to produce a local immunosuppressive environment. Another, which has been studied in the context of islet transplantation are mesenchymal stem cells. These cells’ properties include the release of soluble cytokines with local immunosuppressive impacts as well as growth immunomodulatory effects. Studies have shown improved islet viability and increased levels of immunomodulatory cytokines when co-encapsulated with mesenchymal stem cells, due to the immunosuppressive properties [106,110,122].

Traditionally, secreted cytokines from islets and immune cells were quantified via ELISA, however, flow cytometry is now favoured due to less preparation and assay interpretation time as well as improved cost-effectiveness [126,127,128]. CBA can detect and quantify multiple cytokines, either from biological samples (plasma) or media used to culture cells [126,127,128].

Microencapsulated pancreatic islets and β-cells are capable of taking up nutrients and secreting associated waste products through the semi-permeable membrane of the microencapsulation system [71,73,129,130]. This can be visualized in Figure 1. As a result, under in vitro conditions, their metabolic and bioenergetic profiles can be determined with the use of instrumentation such as the Seahorse extracellular Flux Analyzer [5]. This instrument can detect, via fluorescent sensors, changes in acidity of the media, in either 24 or 96 well plates containing encapsulated cells. The Seahorse can then detect changes in pH due to H^+^ leakage from the encapsulated cells into the surrounding media [131]. This in turn would be proportional to the level of respiration and metabolic activity of the encapsulated cells, and testing can occur under aerobic and anaerobic conditions to understand how encapsulated cells behave and function under both conditions of oxygen deprivation and hypoxia [129,131,132,133].

Furthermore, a range of glucose concentrations can be used to culture and study the encapsulated islets and β-cells, indicating that respiration and metabolic status can be determined across a range of testing conditions [5,132]. The metabolic and respiration measurements are taken in a non-invasive manner to the microcapsules [5]. Furthermore, the data obtained is measured in real time, so it is possible to determine over long durations of study at which time points encapsulated cells begin to malfunction or lose their viability [5,129,131,132,133]. Based on the data obtained, the microcapsule design can be altered to better cater for cellular respiration and metabolic activities and provide further complementary information to pre-determined assays such as MTT viability and secreted insulin content in order to better predict in vivo performance and help develop improved delivery systems [5].

In summary of the Seahorse, each injection vessel contains a compound which can penetrate the microcapsule membrane and alters key cellular respiratory or metabolic functions in order to study how the cells respond to various metabolic and energetic challenges. This in turn gives a more rounded understanding of which pharmaceutical formulations better cater for cellular biological activities and which β-cell line or islet sub-type is better suited for encapsulation and transplantation. Encapsulated cells are placed in culture media (the makeup of which determined by the researchers to better reflect the testing conditions, e.g., varying glucose levels to test how cells respond) and over time these cells release analytes (such as H^+^) which correspond to the degree of encapsulated cellular respiration and metabolism [5,131,132,133].

In 2014, Mooranian et al. were believed to be the first to apply the Seahorse extracellular Flux Analyzer techniques to microencapsulated pancreatic β-cells to determine which pharmaceutical formulations better cater for unique encapsulated cellular activities. This was conducted via measurements including those of cellular respiration and microenergetics, providing information about size, stability, and morphology of their tested microcapsules. Subsequently, the authors were able to determine, using real time data, that certain complex co-formulations of multiple polyelectrolytes-hydrogels mixed with bile acids result in gradual encapsulated β-cell hypoxia with loss of cellular integrity and apoptosis, providing invaluable information [5].

## 5. Conclusions

Outlined within this review are microcapsule analysis, immunogenic considerations, and in vitro studies, including of microencapsulated cells and islets. Overall, large scale experimentation must be carried out on the components of microcapsules, in addition to the resultant microcapsules without cells, and finally the microencapsulated cells. This includes in vitro, ex vivo and in vivo analysis. Within this, the physical and chemical properties must be assessed to determine the most ideal encapsulation system and components in order to offer a functional encapsulated cell delivery system and transplantable microdevice.

## 6. Limitations

The methods outlined within this review allow characterisation and subsequent evaluation, including in vitro, of microcapsules; hence, providing invaluable information. However, as with all research, further evaluation and in vivo studies would be required to progress research, with such analysis beyond the scope of this review.

## Figures and Tables

**Table 1:** Mechanisms, information provided by, advantages and disadvantages of microcapsule analysis techniques.

Technique	Information Provided	Advantages	Disadvantages
Confocal laser scanning microscopy	Analyse distribution of polymers and cross linking ions [11]	Indicates polymer-excipient interactions, and permeability of membranes [12,13]	Phototoxicity of live cells, low speed and sensitivity ratio [14]
Scanning electron microscopy	Visualize and characterize surface structure, geometry and surface composition in detailed 3D images [21,22]	Topography and morphology of the microcapsules [23,24]	Harsh conditions can result in dehydration and deformity, giving rise to artefacts [25]
Electron dispersive X-ray spectroscopy	Analyses surface and elemental characterization of microcapsules [26]	Built-in software provides information on both atomic and weight percentages [26]	Only indicates surface elemental composition, not chemical groups below the surface [28,29,30]
Fourier transform infrared spectroscopy	Provides an absorption spectrum of the sample, indicating the nature of chemical groups on the surface [31,32,33]	Looks at the surface of microcapsules on a micrometre scale [31,32,33]	Samples must be crushed for analysis, destroying them [31]
Differential scanning calorimetry	Indicates physical changes with temperature over time [41]	Assessment of thermal stability and nature of interaction amongst excipients [43]	Bubbles invalidate results and overlapping hydration peaks limit use [44]
Swelling studies	Assess mechanical properties which are important to prevent triggering the immune system [45,46]	Conducted at temperatures and conditions mimicking physiological parameters [27,47,48]	Relies upon weight changes to indicate a swelling index [45,46]
Mechanical Strength	Measures strength of microcapsules under pH or osmotic stresses [51,52]	Allows the number of fractured capsules to be evaluated under physiological conditions stresses [51,52]	Relies on visual inspection and microscopy to compare the broken or damaged capsules to original conditions [51,52]
Surface Charge	Determines the electrokinetic potential [55,56]	Indicates potential interaction between microcapsules and surrounding tissues [55,56]	Must be measured under conditions analogous to the physiological state for accuracy [55,57]

**Table 2:** Mechanisms, information provided by, advantages and disadvantages of Microencapsulated Islets and Cell Line analysis techniques.

Technique	Information Provided	Advantages	Disadvantages
MTT	Photometrically determines cell viability [85,86,87]	Doesn’t require destruction of microcapsules for analysis, improved sensitivity with incubation [85,86,87]	Microcapsules assayed are not viable post analysis and the increased incubation is more cytotoxic. Does not quantify cells or assess cell proliferation [91,95,96]
Trypan blue assay	Cell viability assessment [93,94]	Simple assay by which cells without dye are viable, cells with are non-viable [93,94]	Subjective test, which provides no information on function or growth ability of the cells [93,94]. Microcapsules must be ruptured for analysis [91,92]
Carboxyfluorescein succinimidyl ester staining	Staining of cells’ nuclei for viability assessments via fluorescence microscopy [95,96]	In appropriate concentrations no adverse impacts on the cell occur [97]	In high concentrations the dye is toxic to the cells [98]
BrdU staining and analysis with flow cytometry or ELISA	Determines cell prolideration rates and cell cycle status [101]	BrdU is retained in cells independent of cell cycle phase, allowing detection in post-mitotic cells [100]	Requires strong denaturing conditions, degrading specimens, with staining intensity wholly dependent upon denaturing conditions [103]

## References

[1] Manay P., Turgeon N., Axelrod D.A. (2020). Role of Whole Organ Pancreas Transplantation in the Day of Bioartificial and Artificial Pancreas. Curr. Transplant. Rep..

[2] Wang X. (2019). Bioartificial Organ Manufacturing Technologies. Cell Transplant..

[3] Bünger C., Jahnke A., Stange J., De Vos P., Hopt U. (2002). MTS Colorimetric Assay in Combination with a Live-Dead Assay for Testing Encapsulated L929 Fibroblasts in Alginate Poly-l-Lysine Microcapsules In vitro. Artif. Organs.

[4] Mooranian A., Negrulj R., Jamieson E., Morahan G., Al-Salami H. (2016). Biological Assessments of Encapsulated Pancreatic β-Cells: Their Potential Transplantation in Diabetes. Cell. Mol. Bioeng..

[5] Mooranian A., Negrulj R., Chen-Tan N., Fakhoury M., Arfuso F., Jones F., Al-Salami H. (2014). Advanced bile acid-based multi-compartmental microencapsulated pancreatic beta-cells integrating a polyelectrolyte-bile acid formulation, for diabetes treatment. Artif. Cells Nanomed. Biotechnol..

[6] Krishnan R., Ko D., Foster C.E., Liu W., Smink A.M., de Haan B., De Vos P., Lakey J.R. (2017). Immunological Challenges Facing Translation of Alginate Encapsulated Porcine Islet Xenotransplantation to Human Clinical Trials. Methods Mol. Biol..

[7] Giorno L., De Bartolo L., Drioli E., Moo-Young M. (2011). 2.20—Membrane Bioreactors. Comprehensive Biotechnology.

[8] Orive G., Hernandez R.M., Gascon A.R., Igartua M., Pedraz J.L. (2003). Development and optimisation of alginate-PMCG-alginate microcapsules for cell immobilisation. Int. J. Pharm..

[9] Wagle S.R., Walker D., Kovacevic B., Gedawy A., Mikov M., Golocorbin-Kon S., Mooranian A., Al-Salami H. (2020). Micro-Nano formulation of bile-gut delivery: Rheological, stability and cell survival, basal and maximum respiration studies. Sci. Rep..

[10] Thanos C.G., Calafiore R., Basta G., Bintz B.E., Bell W.J., Hudak J., Vasconcellos A., Schneider P., Skinner S.J., Geaney M. (2007). Formulating the alginate-polyornithine biocapsule for prolonged stability: Evaluation of composition and manufacturing technique. J. Biomed. Mater. Res. A.

[11] Strand B.L., Morch Y.A., Espevik T., Skjak-Braek G. (2003). Visualization of alginate-poly-L-lysine-alginate microcapsules by confocal laser scanning microscopy. Biotechnol. Bioeng..

[12] Kouisni L., Rochefort D. (2009). Confocal microscopy study of polymer microcapsules for enzyme immobilisation in paper substrates. J. Appl. Polym. Sci..

[13] Abdel-Hafez S.M., Hathout R.M., Sammour O.A. (2018). Tracking the transdermal penetration pathways of optimized curcumin-loaded chitosan nanoparticles via confocal laser scanning microscopy. Int. J. Biol. Macromol..

[14] Jonkman J., Brown C.M. (2015). Any Way You Slice It-A Comparison of Confocal Microscopy Techniques. J. Biomol. Tech..

[15] Tang F., Wang C., Wang X., Li L. (2015). Facile Synthesis of Biocompatible Fluorescent Nanoparticles for Cellular Imaging and Targeted Detection of Cancer Cells. ACS Appl. Mater. Interfaces.

[16] Schrinner K., Veiter L., Schmideder S., Doppler P., Schrader M., Münch N., Althof K., Kwade A., Briesen H., Herwig C. (2020). Morphological and physiological characterization of filamentous Lentzea aerocolonigenes: Comparison of biopellets by microscopy and flow cytometry. PLoS ONE.

[17] Truernit E., Haseloff J. (2008). A simple way to identify non-viable cells within living plant tissue using confocal microscopy. Plant Methods.

[18] Klak M., Kowalska P., Dobrzański T., Tymicki G., Cywoniuk P., Gomółka M., Kosowska K., Bryniarski T., Berman A., Dobrzyń A. (2021). Bionic Organs: Shear Forces Reduce Pancreatic Islet and Mammalian Cell Viability during the Process of 3D Bioprinting. Micromachines.

[19] Monte M.J., Marin J.J., Antelo A., Vazquez-Tato J. (2009). Bile acids: Chemistry, physiology, and pathophysiology. World J. Gastroenterol WJG.

[20] Faustino C., Serafim C., Rijo P., Reis C.P. (2016). Bile acids and bile acid derivatives: Use in drug delivery systems and as therapeutic agents. Expert Opin. Drug Deliv..

[21] Al-Kassas R.S., Al-Gohary O.M., Al-Faadhel M.M. (2007). Controlling of systemic absorption of gliclazide through incorporation into alginate beads. Int. J. Pharm..

[22] Ajun W., Yan S., Li G., Huili L. (2009). Preparation of aspirin and probucol in combination loaded chitosan nanoparticles and in vitro release study. Carbohydr. Polym..

[23] Prajapati S.K., Tripathi P., Ubaidulla U., Anand V. (2008). Design and development of gliclazide mucoadhesive microcapsules: In vitro and in vivo evaluation. AAPS PharmSciTech.

[24] Mooranian A., Negrulj R., Chen-Tan N., Al-Sallami H.S., Fang Z., Mukkur T., Mikov M., Golocorbin-Kon S., Fakhoury M., Watts G.F. (2014). Microencapsulation as a novel delivery method for the potential antidiabetic drug, Probucol. Des. Dev. Ther..

[25] Rokstad A.M.A., Lacík I., de Vos P., Strand B.L., Rokstad A.M.A., Lacík I., de Vos P., Strand B.L. (2014). Advances in biocompatibility and physico-chemical characterization of microspheres for cell encapsulation. Adv. Drug. Deliv. Rev..

[26] Khan M.S.I., Oh S.-W., Kim Y.-J. (2020). Power of Scanning Electron Microscopy and Energy Dispersive X-Ray Analysis in Rapid Microbial Detection and Identification at the Single Cell Level. Sci. Rep..

[27] Negrulj R., Mooranian A., Chen-Tan N., Al-Sallami H.S., Mikov M., Golocorbin-Kon S., Fakhoury M., Watts G.F., Arfuso F., Al-Salami H. (2015). Swelling, mechanical strength, and release properties of probucol microcapsules with and without a bile acid, and their potential oral delivery in diabetes. Artif. Cells Nanomed. Biotechnol..

[28] Awasthi R., Kulkarni G.T. (2014). Development of novel gastroretentive drug delivery system of gliclazide: Hollow beads. Drug Dev. Ind. Pharm..

[29] Slater T.J.A., Camargo P.H.C., Burke M.G., Zaluzec N.J., Haigh S.J. (2014). Understanding the limitations of the Super-X energy dispersive x-ray spectrometer as a function of specimen tilt angle for tomographic data acquisition in the S/TEM. J. Phys. Conf. Ser..

[30] Newbury D.E., Ritchie N.W.M. (2013). Is Scanning Electron Microscopy/Energy Dispersive X-ray Spectrometry (SEM/EDS) Quantitative?. Scanning.

[31] Margariti C. (2019). The application of FTIR microspectroscopy in a non-invasive and non-destructive way to the study and conservation of mineralised excavated textiles. Herit. Sci..

[32] Vogt S., Löffler K., Dinkelacker A.G., Bader B., Autenrieth I.B., Peter S., Liese J. (2019). Fourier-Transform Infrared (FTIR) Spectroscopy for Typing of Clinical Enterobacter cloacae Complex Isolates. Front. Microbiol..

[33] Mathavan S., Chen-Tan N., Arfuso F., Al-Salami H. (2016). The role of the bile acid chenodeoxycholic acid in the targeted oral delivery of the anti-diabetic drug gliclazide, and its applications in type 1 diabetes. Artif. Cells Nanomed. Biotechnol..

[34] van Hoogmoed C.G., Busscher H.J., de Vos P. (2003). Fourier transform infrared spectroscopy studies of alginate-PLL capsules with varying compositions. J. Biomed. Mater. Res. A.

[35] Azadi S.A., Vasheghani-Farahani E., Hashemi-Najafbabadi S., Godini A. (2016). Co-encapsulation of pancreatic islets and pentoxifylline in alginate-based microcapsules with enhanced immunosuppressive effects. Prog. Biomater..

[36] Elizondo D.M., Brandy N.Z.D., da Silva R.L.L., de Moura T.R., Ali J., Yang D., Lipscomb M.W. (2020). Pancreatic islets seeded in a novel bioscaffold forms an organoid to rescue insulin production and reverse hyperglycemia in models of type 1 diabetes. Sci. Rep..

[37] Tam S.K., Dusseault J., Polizu S., Ménard M., Hallé J.-P., Yahia L.H. (2005). Physicochemical model of alginate–poly-L-lysine microcapsules defined at the micrometric/nanometric scale using ATR-FTIR, XPS, and ToF-SIMS. Biomaterials.

[38] Tam S., Bilodeau S., Dusseault J., Langlois G., Hallé J.-P., Yahia L. (2011). Biocompatibility and physicochemical characteristics of alginate–polycation microcapsules. Acta Biomater..

[39] Ehrhart F., Mettler E., Böse T., Weber M.M., Vásquez J.A., Zimmermann H. (2013). Biocompatible Coating of Encapsulated Cells Using Ionotropic Gelation. PLoS ONE.

[40] Chiappe C., Pomelli C.S., Sartini S. (2019). Combined Use of Scanning Electron Microscopy–Energy-Dispersive X-ray Spectroscopy and Fourier Transform Infrared Imaging Coupled with Principal Component Analysis in the Study of Ancient Egyptian Papyri. ACS Omega.

[41] Gill P., Moghadam T.T., Ranjbar B. (2010). Differential scanning calorimetry techniques: Applications in biology and nanoscience. J. Biomol. Tech. JBT.

[42] Takka S., Çali A.G. (2010). Bile salt-reinforced alginate-chitosan beads. Pharm. Dev. Technol..

[43] Awasthi R., Kulkarni G.T. (2012). Development of novel gastroretentive floating particulate drug delivery system of gliclazide. Curr. Drug Deliv..

[44] Chiu M.H., Prenner E.J. (2011). Differential scanning calorimetry: An invaluable tool for a detailed thermodynamic characterization of macromolecules and their interactions. J. Pharm. Bioallied. Sci..

[45] Mooranian A., Negrulj R., Al-Sallami H.S., Fang Z., Mikov M., Golocorbin-Kon S., Fakhoury M., Arfuso F., Al-Salami H. (2015). Release and swelling studies of an innovative antidiabetic-bile acid microencapsulated formulation, as a novel targeted therapy for diabetes treatment. J. Microencapsul..

[46] Paredes Juárez G.A., Spasojevic M., Faas M.M., de Vos P. (2014). Immunological and technical considerations in application of alginate-based microencapsulation systems. Front. Bioeng. Biotechnol..

[47] Singh B., Sharma D.K., Gupta A. (2009). The controlled and sustained release of a fungicide from starch and alginate beads. J. Environ. Sci. Health Part B.

[48] Mooranian A., Negrulj R., Mathavan S., Martinez J., Sciarretta J., Chen-Tan N., Mukkur T.K., Mikov M., Lalic-Popovic M., Stojančević M. (2014). Stability and Release Kinetics of an Advanced Gliclazide-Cholic Acid Formulation: The Use of Artificial-Cell Microencapsulation in Slow Release Targeted Oral Delivery of Antidiabetics. J. Pharm. Innov..

[49] Ahmad Raus R., Wan Nawawi W.M.F., Nasaruddin R.R. (2020). Alginate and alginate composites for biomedical applications. Asian J. Pharm. Sci..

[50] Chan G., Mooney D.J. (2013). Ca(2+) released from calcium alginate gels can promote inflammatory responses in vitro and in vivo. Acta Biomater..

[51] Jiin W.Y. (2000). Development of new polycations for cell encapsulation with alginate. Mater. Sci. Eng..

[52] Mathavan S., Chen-Tan N., Arfuso F., Al-Salami H. (2016). A comprehensive study of novel microcapsules incorporating gliclazide and a permeation enhancing bile acid: Hypoglycemic effect in an animal model of Type-1 diabetes. Drug Deliv..

[53] Lengyel M., Kállai-Szabó N., Antal V., Laki A.J., Antal I. (2019). Microparticles, Microspheres, and Microcapsules for Advanced Drug Delivery. Sci. Pharm..

[54] Zhao H., Fei X., Cao L., Zhang B., Liu X. (2019). Relation between the particle size and release characteristics of aromatic melamine microcapsules in functional textile applications. RSC Adv..

[55] de Vos P., de Haan B.J., Kamps J.A.A.M., Faas M.M., Kitano T. (2007). Zeta-potentials of alginate-PLL capsules: A predictive measure for biocompatibility?. J. Biomed. Mater. Res. Part A.

[56] Mooranian A., Negrulj R., Takechi R., Jamieson E., Morahan G., Al-Salami H. (2017). Electrokinetic potential-stabilization by bile acid-microencapsulating formulation of pancreatic β-cells cultured in high ratio poly-L-ornithine-gel hydrogel colloidal dispersion: Applications in cell-biomaterials, tissue engineering and biotechnological applications. Artif Cells Nanomed Biotechnol.

[57] de Vos P., Spasojevic M., de Haan B.J., Faas M.M. (2012). The association between in vivo physicochemical changes and inflammatory responses against alginate based microcapsules. Biomaterials.

[58] Mooranian A., Negrulj R., Al-Sallami H.S., Fang Z., Mikov M., Golocorbin-Kon S., Fakhoury M., Watts G.F., Matthews V., Arfuso F. (2014). Probucol Release from Novel Multicompartmental Microcapsules for the Oral Targeted Delivery in Type 2 Diabetes. AAPS PharmSciTech.

[59] Mooranian A., Negrulj R., Arfuso F., Al-Salami H. (2014). Characterization of a novel bile acid-based delivery platform for microencapsulated pancreatic β-cells. Artif. Cells Nanomed. Biotechnol..

[60] Lacík I. (2006). Polymer chemistry in diabetes treatment by encapsulated islets of Langerhans: Review to 2006. Aust. J. Chem..

[61] Rickels M.R., Robertson R.P. (2019). Pancreatic Islet Transplantation in Humans: Recent Progress and Future Directions. Endocr. Rev..

[62] Teramura Y., Iwata H. (2010). Bioartificial pancreas microencapsulation and conformal coating of islet of Langerhans. Adv. Drug Deliv. Rev..

[63] Aijaz A., Vaninov N., Allen A., Barcia R.N., Parekkadan B. (2019). Convergence of Cell Pharmacology and Drug Delivery. STEM CELLS Transl. Med..

[64] Thanos C.G., Bintz B.E., Emerich D.F. (2007). Stability of alginate-polyornithine microcapsules is profoundly dependent on the site of transplantation. J. Biomed. Mater. Res. A.

[65] Kelly P., Maguire P.B., Bennett M., Fitzgerald D.J., Edwards R.J., Thiede B., Treumann A., Collins J.K., O’Sullivan G.C., Shanahan F. (2005). Correlation of probiotic Lactobacillus salivarius growth phase with its cell wall-associated proteome. FEMS Microbiol. Lett..

[66] De Vos P., Hamel A., Tatarkiewicz K. (2002). Considerations for successful transplantation of encapsulated pancreatic islets. Diabetologia.

[67] Pareta R.A., Farney A.C., Opara E.C. (2013). Design of a bioartificial pancreas. Pathobiol. J. Immunopathol. Mol. Cell. Biol..

[68] Smink A.M., Faas M.M., de Vos P. (2013). Toward engineering a novel transplantation site for human pancreatic islets. Diabetes.

[69] Pareta R., McQuilling J.P., Farney A., Opara E.C. (2012). Bioartificial pancreas: Evaluation of crucial barriers to clinical application. Organ Donation Transplant. Public Policy Clin. Perspect..

[70] Meier R.P., Seebach J.D., Morel P., Mahou R., Borot S., Giovannoni L., Parnaud G., Montanari E., Bosco D., Wandrey C. (2014). Survival of Free and Encapsulated Human and Rat Islet Xenografts Transplanted into the Mouse Bone Marrow. PLoS ONE.

[71] Murua A., Portero A., Orive G., Hernández R.M., de Castro M., Pedraz J.L. (2008). Cell microencapsulation technology: Towards clinical application. J. Control. Release.

[72] Scharp D.W., Marchetti P. (2013). Encapsulated islets for diabetes therapy: History, current progress, and critical issues requiring solution. Adv. Drug Deliv. Rev..

[73] Steele J., Hallé J.-P., Poncelet D., Neufeld R. (2014). Therapeutic cell encapsulation techniques and applications in diabetes. Adv. Drug Deliv. Rev..

[74] de Groot M., Leuvenink H.G., Keizer P.P., Fekken S., Schuurs T.A., van Schilfgaarde R. (2003). Effective removal of alginate-poly-L-lysine microcapsules from pancreatic islets by use of trypsin–EDTA. J. Biomed. Mater. Res. Part A.

[75] Foster J.L., Williams G., Williams L.J., Tuch B.E. (2007). Differentiation of transplanted microencapsulated fetal pancreatic cells. Transplantation.

[76] Daoud J., Rosenberg L., Tabrizian M. (2010). Pancreatic islet culture and preservation strategies: Advances, challenges, and future outlook. Cell Transplant..

[77] Noguchi H., Miyagi-Shiohira C., Kurima K., Kobayashi N., Saitoh I., Watanabe M., Noguchi Y., Matsushita M. (2015). Islet Culture/Preservation Before Islet Transplantation. Cell Med.

[78] Lin J.-Y., Cheng J., Du Y.-Q., Pan W., Zhang Z., Wang J., An J., Yang F., Xu Y.-F., Lin H. (2020). In vitro expansion of pancreatic islet clusters facilitated by hormones and chemicals. Cell Discov..

[79] Cheng K., Delghingaro-Augusto V., Nolan C.J., Turner N., Hallahan N., Andrikopoulos S., Gunton J.E. (2012). High passage MIN6 cells have impaired insulin secretion with impaired glucose and lipid oxidation. PLoS ONE.

[80] Vaithilingam V., Oberholzer J., Guillemin G.J., Tuch B.E. (2010). The humanized NOD/SCID mouse as a preclinical model to study the fate of encapsulated human islets. Rev. Diabet. Stud..

[81] Ravassard P., Hazhouz Y., Pechberty S., Bricout-Neveu E., Armanet M., Czernichow P., Scharfmann R. (2011). A genetically engineered human pancreatic β cell line exhibiting glucose-inducible insulin secretion. J. Clin. Investig..

[82] Li X., Liu T., Song K., Yao L., Ge D., Bao C., Ma X., Cui Z. (2006). Culture of neural stem cells in calcium alginate beads. Biotechnol. Prog..

[83] Riss T.L., Moravec R.A., Niles A.L., Duellman S., Benink H., Worzella T.J., Minor L., Morkossian S., Grossman A. (2013). Cell viability assays. Assay Guidance Manual.

[84] Sasaki T., Tamaki J., Nishizawa K., Kojima T., Tanaka R., Moriya R., Sasaki H., Maruyama H. (2019). Evaluation of cell viability and metabolic activity of a 3D cultured human epidermal model using a dynamic autoradiographic technique with a PET radiopharmaceutical. Sci. Rep..

[85] Mooranian A., Negrulj R., Al-Salami H. (2016). The incorporation of water-soluble gel matrix into bile acid-based microcapsules for the delivery of viable β-cells of the pancreas, in diabetes treatment: Biocompatibility and functionality studies. Drug Deliv. Transl. Res..

[86] Mooranian A., Negrulj R., Al-Salami H. (2016). The Influence of Stabilized Deconjugated Ursodeoxycholic Acid on Polymer-Hydrogel System of Transplantable NIT-1 Cells. Pharm. Res..

[87] Riss T.L., Moravec R.A., Niles A.L., Duellman S., Benink H.A., Worzella T.J., Minor L. (2016). Cell Viability Assays.

[88] Scarcello E., Lambremont A., Vanbever R., Jacques P.J., Lison D. (2020). Mind your assays: Misleading cytotoxicity with the WST-1 assay in the presence of manganese. PLoS ONE.

[89] Koyanagi M., Kawakabe S., Arimura Y. (2016). A comparative study of colorimetric cell proliferation assays in immune cells. Cytotechnology.

[90] Xiao J., Zhang Y., Wang J., Yu W., Wang W., Ma X. (2010). Monitoring of Cell Viability and Proliferation in Hydrogel-Encapsulated System by Resazurin Assay. Appl. Biochem. Biotechnol..

[91] Werner M., Biss K., Jérôme V., Hilbrig F., Freitag R., Zambrano K., Hübner H., Buchholz R., Mahou R., Wandrey C. (2013). Use of the mitochondria toxicity assay for quantifying the viable cell density of microencapsulated jurkat cells. Biotechnol. Prog..

[92] Mooranian A., Negrulj R., Al-Salami H., Morahan G., Jamieson E. (2016). Designing anti-diabetic β-cells microcapsules using polystyrenic sulfonate, polyallylamine, and a tertiary bile acid: Morphology, bioenergetics, and cytokine analysis. Biotechnol. Prog..

[93] Strober W. (2015). Trypan Blue Exclusion Test of Cell Viability. Curr. Protoc. Immunol..

[94] Piccinini F., Tesei A., Arienti C., Bevilacqua A. (2017). Cell Counting and Viability Assessment of 2D and 3D Cell Cultures: Expected Reliability of the Trypan Blue Assay. Biol. Proced. Online.

[95] Mooranian A., Negrulj R., Takechi R., Jamieson E., Morahan G., Al-Salami H. (2018). Influence of Biotechnological Processes, Speed of Formulation Flow and Cellular Concurrent Stream-Integration on Insulin Production from β-cells as a Result of Co-Encapsulation with a Highly Lipophilic Bile Acid. Cell Mol. Bioeng..

[96] Zavala G., Ramos M.-P., Figueroa-Valdés A.I., Cisternas P., Wyneken U., Hernández M., Toa P., Salmons B., Dangerfield J., Gunzburg W.H. (2020). Semipermeable Cellulose Beads Allow Selective and Continuous Release of Small Extracellular Vesicles (sEV) From Encapsulated Cells. Front. Pharmacol..

[97] Banks H.T., Sutton K.L., Clayton Thompson W., Bocharov G., Roose D., Schenkel T., Meyerhans A. (2011). Estimation of Cell Proliferation Dynamics Using CFSE Data. Bull. Math. Biol..

[98] Lašťovička J., Rataj M., Bartůňková J. (2016). Assessment of lymphocyte proliferation for diagnostic purpose: Comparison of CFSE staining, Ki-67 expression and 3H-thymidine incorporation. Hum. Immunol..

[99] Chan K.W.Y., Liu G., Song X., Kim H., Yu T., Arifin D.R., Gilad A.A., Hanes J., Walczak P., van Zijl P.C.M. (2013). MRI-detectable pH nanosensors incorporated into hydrogels for in vivo sensing of transplanted-cell viability. Nat. Mater..

[100] Sauerzweig S., Baldauf K., Braun H., Reymann K.G. (2009). Time-dependent segmentation of BrdU-signal leads to late detection problems in studies using BrdU as cell label or proliferation marker. J. Neurosci. Methods.

[101] Padet L., St-Amour I., Aubin E., Proulx D.P., Bazin R., Lemieux R. (2009). Dose-dependent inhibition of BrdU detection in the cell proliferation ELISA by culture medium proteins. J. Immunoass. Immunochem..

[102] Pang L., Reddy P.V., McAuliffe C.I., Colvin G., Quesenberry P.J. (2003). Studies on BrdU labeling of hematopoietic cells: Stem cells and cell lines. J. Cell. Physiol..

[103] Salic A., Mitchison T.J. (2008). A chemical method for fast and sensitive detection of DNA synthesis in vivo. Proc. Natl. Acad. Sci. USA.

[104] Kedziorek D.A., Hofmann L.V., Fu Y., Gilson W.D., Cosby K.M., Kohl B., Barnett B.P., Simons B.W., Walczak P., Bulte J.W. (2012). X-ray-visible microcapsules containing mesenchymal stem cells improve hind limb perfusion in a rabbit model of peripheral arterial disease. Stem Cells.

[105] Fooks L.J., Gibson G.R. (2002). In vitro investigations of the effect of probiotics and prebiotics on selected human intestinal pathogens. FEMS Microbiol. Ecol..

[106] Vaithilingam V., Evans M.D.M., Lewy D.M., Bean P.A., Bal S., Tuch B.E. (2017). Co-encapsulation and co-transplantation of mesenchymal stem cells reduces pericapsular fibrosis and improves encapsulated islet survival and function when allografted. Sci. Rep..

[107] Hårdstedt M., Lindblom S., Hong J., Nilsson B., Korsgren O., Ronquist G. (2015). A novel model for studies of blood-mediated long-term responses to cellular transplants. Upsala J. Med Sci..

[108] Ekdahl K.N., Hong J., Hamad O.A., Larsson R., Nilsson B., Lambris J.D., Holers V.M., Ricklin D. (2013). Evaluation of the Blood Compatibility of Materials, Cells, and Tissues: Basic Concepts, Test Models, and Practical Guidelines. Complement Therapeutics.

[109] Hårdstedt M., Lindblom S., Karlsson-Parra A., Nilsson B., Korsgren O. (2016). Characterization of Innate Immunity in an Extended Whole Blood Model of Human Islet Allotransplantation. Cell Transplant..

[110] Anderson J.M. (2016). Future challenges in the in vitro and in vivo evaluation of biomaterial biocompatibility. Regen. Biomater..

[111] Bernard M., Jubeli E., Pungente M.D., Yagoubi N. (2018). Biocompatibility of polymer-based biomaterials and medical devices—Regulations, in vitro screening and risk-management. Biomater. Sci..

[112] Chang D.T., Jones J.A., Meyerson H., Colton E., Kwon I.K., Matsuda T., Anderson J.M. (2008). Lymphocyte/macrophage interactions: Biomaterial surface-dependent cytokine, chemokine, and matrix protein production. J. Biomed. Mater. Res. A.

[113] Donath M.Y., Böni-Schnetzler M., Ellingsgaard H., Halban P.A., Ehses J.A. (2010). Cytokine production by islets in health and diabetes: Cellular origin, regulation and function. Trends Endocrinol. Metab..

[114] Viana K.F., Fiuza J.A., Gannavaram S., Dey R., Selvapandiyan A., Bartholomeu D.C., da Silveira-Lemos D., Bueno L.L., Dutra W.O., Fujiwara R.T. (2016). Application of rapid in vitro co-culture system of macrophages and T-cell subsets to assess the immunogenicity of dogs vaccinated with live attenuated Leishmania donovani centrin deleted parasites (LdCen^−/−^). Parasit. Vectors.

[115] Luukkainen A., Puan K.J., Yusof N., Lee B., Tan K.S., Liu J., Yan Y., Toppila-Salmi S., Renkonen R., Chow V.T. (2018). A Co-culture Model of PBMC and Stem Cell Derived Human Nasal Epithelium Reveals Rapid Activation of NK and Innate T Cells Upon Influenza A Virus Infection of the Nasal Epithelium. Front. Immunol..

[116] Barshes N.R., Wyllie S., Goss J.A. (2005). Inflammation-mediated dysfunction and apoptosis in pancreatic islet transplantation: Implications for intrahepatic grafts. J. Leukoc. Biol..

[117] Wilson J.T., Chaikof E.L. (2008). Challenges and emerging technologies in the immunoisolation of cells and tissues. Adv. Drug Deliv. Rev..

[118] Vaithilingam V., Bal S., Tuch B.E. (2017). Encapsulated Islet Transplantation: Where Do We Stand?. Rev. Diabet. Stud..

[119] Lee Y.Y., Hong S.H., Lee Y.J., Chung S.S., Jung H.S., Park S.G., Park K.S. (2010). Tauroursodeoxycholate (TUDCA), chemical chaperone, enhances function of islets by reducing ER stress. Biochem. Biophys. Res. Commun..

[120] Maedler K., Sergeev P., Ris F., Oberholzer J., Joller-Jemelka H.I., Spinas G.A., Kaiser N., Halban P.A., Donath M.Y. (2002). Glucose-induced β cell production of IL-1β contributes to glucotoxicity in human pancreatic islets. J. Clin. Investig..

[121] Piemonti L., Leone B.E., Nano R., Saccani A., Monti P., Maffi P., Bianchi G., Sica A., Peri G., Melzi R. (2002). Human pancreatic islets produce and secrete MCP-1/CCL2: Relevance in human islet transplantation. Diabetes.

[122] Negrulj R., Mooranian A., Al-Salami H. (2013). Potentials and Limitations of Bile Acids in Type 2 Diabetes Mellitus: Applications of Microencapsulation as a Novel Oral Delivery System. J. Endocrinol. Diabetes Mellit..

[123] Su J., Hu B.-H., Lowe W.L., Kaufman D.B., Messersmith P.B. (2010). Anti-inflammatory peptide-functionalized hydrogels for insulin-secreting cell encapsulation. Biomaterials.

[124] Nishizawa M., Saigusa M., Saeki H. (2016). Conjugation with alginate oligosaccharide via the controlled Maillard reaction in a dry state is an effective method for the preparation of salmon myofibrillar protein with excellent anti-inflammatory activity. Fish. Sci..

[125] Neves M.I., Moroni L., Barrias C.C. (2020). Modulating Alginate Hydrogels for Improved Biological Performance as Cellular 3D Microenvironments. Front. Bioeng. Biotechnol..

[126] Koss M.J., Pfister M., Koch F.H. (2011). Inflammatory and angiogenic protein detection in the human vitreous: Cytometric bead assay. J. Ophthalmol..

[127] Ichikawa R., Takayama T., Yoneno K., Kamada N., Kitazume M.T., Higuchi H., Matsuoka K., Watanabe M., Itoh H., Kanai T. (2012). Bile acids induce monocyte differentiation toward interleukin-12 hypo-producing dendritic cells via a TGR5-dependent pathway. Immunology.

[128] Maier R., Weger M., Haller-Schober E.-M., El-Shabrawi Y., Theisl A., Barth A., Aigner R., Haas A. (2006). Application of multiplex cytometric bead array technology for the measurement of angiogenic factors in the vitreous. Mol. Vis..

[129] Gerencser A.A., Neilson A., Choi S.W., Edman U., Yadava N., Oh R.J., Ferrick D.A., Nicholls D.G., Brand M.D. (2009). Quantitative microplate-based respirometry with correction for oxygen diffusion. Anal. Chem..

[130] Weir G.C. (2013). Islet encapsulation: Advances and obstacles. Diabetologia.

[131] Wu M., Neilson A., Swift A.L., Moran R., Tamagnine J., Parslow D., Armistead S., Lemire K., Orrell J., Teich J. (2007). Multiparameter metabolic analysis reveals a close link between attenuated mitochondrial bioenergetic function and enhanced glycolysis dependency in human tumor cells. Am. J. Physiol. Cell Physiol..

[132] Wikstrom J.D., Sereda S.B., Stiles L., Elorza A., Allister E.M., Neilson A., Ferrick D.A., Wheeler M.B., Shirihai O.S. (2012). A novel high-throughput assay for islet respiration reveals uncoupling of rodent and human islets. PLoS ONE.

[133] Brand M., Nicholls D. (2011). Assessing mitochondrial dysfunction in cells. Biochem. J..

